# Development and validation of a machine learned algorithm to IDENTIFY functionally significant coronary artery disease

**DOI:** 10.3389/fcvm.2022.956147

**Published:** 2022-09-02

**Authors:** Thomas Stuckey, Frederick Meine, Thomas McMinn, Jeremiah P. Depta, Brett Bennett, Thomas McGarry, William Carroll, David Suh, John A. Steuter, Michael Roberts, Horace R. Gillins, Emmanuel Lange, Farhad Fathieh, Timothy Burton, Ali Khosousi, Ian Shadforth, William E. Sanders, Mark G. Rabbat

**Affiliations:** ^1^Cone Health Heart and Vascular Center, Greensboro, NC, United States; ^2^Novant Health New Hanover Regional Medical Center, Wilmington, NC, United States; ^3^Austin Heart, Austin, TX, United States; ^4^Rochester General Hospital, Rochester, NY, United States; ^5^Jackson Heart Clinic, Jackson, MS, United States; ^6^Oklahoma Heart Hospital, Oklahoma City, OK, United States; ^7^Cardiology Associates of North Mississippi, Tupelo, MS, United States; ^8^Atlanta Heart Specialists, Atlanta, GA, United States; ^9^Bryan Heart, Lincoln, NE, United States; ^10^Lexington Medical Center, West Columbia, SC, United States; ^11^CorVista Health, Inc., Washington, DC, United States; ^12^CorVista Health, Inc., Analytics For Life Inc., d.b.a CorVista Health, Toronto, ON, Canada; ^13^Loyola University Medical Center, Maywood, IL, United States

**Keywords:** coronary artery disease, artificial intelligence, front line testing, machine learning (ML), digital health

## Abstract

**Introduction:**

Multiple trials have demonstrated broad performance ranges for tests attempting to detect coronary artery disease. The most common test, SPECT, requires capital-intensive equipment, the use of radionuclides, induction of stress, and time off work and/or travel. Presented here are the development and clinical validation of an office-based machine learned algorithm to identify functionally significant coronary artery disease without radiation, expensive equipment or induced patient stress.

**Materials and methods:**

The IDENTIFY trial (NCT03864081) is a prospective, multicenter, non-randomized, selectively blinded, repository study to collect acquired signals paired with subject meta-data, including outcomes, from subjects with symptoms of coronary artery disease. Time synchronized orthogonal voltage gradient and photoplethysmographic signals were collected for 230 seconds from recumbent subjects at rest within seven days of either left heart catheterization or coronary computed tomography angiography. Following machine learning on a proportion of these data (*N* = 2,522), a final algorithm was selected, along with a pre-specified cut point on the receiver operating characteristic curve for clinical validation. An unseen set of subject signals (*N* = 965) was used to validate the algorithm.

**Results:**

At the pre-specified cut point, the sensitivity for detecting functionally significant coronary artery disease was 0.73 (95% CI: 0.68–0.78), and the specificity was 0.68 (0.62–0.74). There exists a point on the receiver operating characteristic curve at which the negative predictive value is the same as coronary computed tomographic angiography, 0.99, assuming a disease incidence of 0.04, yielding sensitivity of 0.89 and specificity of 0.42. Selecting a point at which the positive predictive value is maximized, 0.12, yields sensitivity of 0.39 and specificity of 0.88.

**Conclusion:**

The performance of the machine learned algorithm presented here is comparable to common tertiary center testing for coronary artery disease. Employing multiple cut points on the receiver operating characteristic curve can yield the negative predictive value of coronary computed tomographic angiography and a positive predictive value approaching that of myocardial perfusion imaging. As such, a system employing this algorithm may address the need for a non-invasive, no radiation, no stress, front line test, and hence offer significant advantages to the patient, their physician, and healthcare system.

## Introduction

Functionally significant coronary artery disease (CAD) is defined as a reduction in blood flow using fractional flow reserve (FFR, evaluating the impact of a lesion on blood flow) of <0.80, an instantaneous wave-free ratio (iFR, another functional flow measure) <0.89, or when both are unavailable, the anatomical presence of at least one lesion in the coronary arteries causing a stenosis of >70% (1). When this definition is met, the patient is referred to as CAD + (i.e., presence of significant CAD), and when not met, CAD- (i.e., absence of significant CAD). Ischemic heart disease (IHD), the manifestation of CAD, continues to be a major public health problem with 1 in 3 adults in the United States (approximately 81 million) experiencing some form of cardiovascular disease, and more than 17 million having CAD. Angina pectoris, affecting 10 million people, is the first symptom of IHD in nearly 50% of patients (2). In recent years, survival rates for patients with IHD have improved, but IHD remains responsible for >350,000 deaths per year and over 1.5 million patients suffer myocardial infarction (1). Clearly, chest pain is ubiquitous with studies showing a lifetime prevalence of 20–40% in the general population (3).

When a patient presents with chest pain, the clinician must determine whether the probability of CAD warrants diagnostic testing, which most commonly entails either a functional or anatomical test. Functional testing options are typically exercise electrocardiography (ECG), nuclear stress testing with a single photon emission computed tomography (SPECT) or stress echocardiography (1). Anatomical testing using coronary computed tomography angiography (coronary CTA) has also emerged as a viable testing modality (4). Based on the results of diagnostic testing, in combination with the patient’s pre-test probability, the clinician may choose to proceed further in the CAD diagnostic pathway to invasive angiography, which is the gold standard test for assessment of CAD. Invasive angiography involves cardiac catheterization under fluoroscope imaging, accompanied with a contrast agent to image the coronary arteries.

To better understand CAD diagnostic testing modalities, the PROMISE (4) study examined the outcomes associated with 10,003 patients referred to either functional testing using the options previously listed, or anatomical testing with coronary CTA. During the 2-year follow-up period, the primary endpoint of death, myocardial infarction, hospitalization for unstable angina, or major procedural complication occurred in 3.3% in the functional arm and 3.0% in the anatomical arm. Despite the similarity in outcome rates, differences emerge in invasive angiography, where 12% of patients in the coronary CTA arm were referred to invasive angiography, as compared to 8.1% in the functional arm. Further, the CAD + rate was higher in coronary CTA as compared to functional, both as a proportion of those referred to invasive angiography (72 vs. 47%) as well as the overall population (8 vs. 4%), indicating that coronary CTA may be more effective at referring patients with disease to invasive angiography. Note that PROMISE uses a CAD definition of a 50% lesion, rather than the 70% prescribed by American College of Cardiology (ACC) guidelines (1), so the rates per ACC would be lower across both coronary CTA and functional testing. The higher CAD detection rate for coronary CTA compared to SPECT based on invasive angiography results shows that, in PROMISE, coronary CTA appears to have higher sensitivity for CAD as compared to functional testing. This observation agrees with recent meta-analysis, which also showed a corresponding superior negative likelihood ratio for coronary CTA (5). Therefore, coronary CTA can more reliably be used as a rule-out test as compared to functional stress testing.

Despite the high negative predictive value of coronary CTA, functional testing remains popular, with nearly 4 million stress tests performed annually in the United States for new-onset, stable chest pain; all of which carry some risks. Maximal capacity exercise testing (as assessed by the patient’s predicted maximal heart rate) is associated with a low but definable incidence of myocardial infarction (MI), cardiac arrest, and death. The use of pharmacologic agents to induce stress has a small risk of drug related adverse events. Coronary CTA is not without its disadvantages, with both coronary CTA and SPECT employing ionizing radiation. With each of these tests, the patient incurs substantial cost, experiences inconveniences, and endures significant time loss. Neither test is universally available, especially in low resource settings. There is a need for a performant, point-of-care, and minimal risk first line method for assessing CAD.

Machine learning holds the promise of delivering such an assessment and there is extensive prior research in this area. In a recent review of AI in the cardiovascular space, Lopez-Jiminez et al. provide an introduction into advancements being made in this area (6). In particular, they identify the single most important aspect of the development of a machine learned model as being the data set used for training and testing as “…*all the ‘rules’ are being created from the data*.” The collection of a dataset that accurately represents the population in which the resultant algorithm will be used, which captures the entire range of disease, and does not introduce bias due to geographic, demographic, or site-specific disparities is therefore crucial to developing and demonstrating the performance of machine learned algorithms.

The availability of large imaging datasets has, in particular, enabled the development of deep learning based models for CAD assessment. One key advantage of deep learning is that the use of multiple layers in the network allows for the internal discovery and utilization of features, as opposed to having to define these ahead of time. Consequently, the features discovered and used are not limited to those that can be conceived of by the researchers by analysis of the problem space. Promising results have been reported in the application of deep learning when assessing CAD based on SPECT images (7), as well as the use of convolutional neural networks to discover features in cardiac magnetic resonance imaging for subsequent use in random forest experiments (8). However, it is often difficult to collect large volumes of high quality clinical data, especially when new devices supply the input for machine learning. When data availability is limited, more traditional machine learning approaches based on manually created features extracted from the available data can be performed. Examples of this include the assessment of CAD using SPECT-derived metrics with support vector machines (9), as well as the use of similar SPECT-derived metrics along with various demographic data used to create a boosted ensemble (Logit-boost) algorithm (10).

Alizadehsani et al., recently published an overview of 256 papers aiming to assess for coronary artery disease utilizing machine learning techniques (11). The various machine learning methods were trained using a variety of input data, including ECG, echocardiograms, demographic data, clinical history information, and laboratory results. As this review demonstrates, frequently the performance of machine learned algorithms is presented based on cross-validation rather than applying the algorithm to an entirely new test set. Whilst cross-validation is a useful way to compare the performance of different machine learning methods across the same dataset, the method is not immune to overfitting (12). Hence, testing a machine learned model against a naïve test set, ideally strictly controlled to not be available to the research team, is considered the gold standard for algorithm performance testing. A recent publication outlining guiding principles for good machine learning practices from the U.S. Food and Drug Administration (FDA), Health Canada, and the United Kingdom’s Medicines and Healthcare products Regulatory Agency (MHRA) highlights the need for such separate, independent, training and test sets, wherein “*…[all potential sources of dependence, including patient, data acquisition, and site factors, are considered and addressed to assure independence*” (13).

This paper presents the results of the clinical performance assessment of a novel, machine-learned algorithm for the determination of significant CAD in a patient at rest using a naïve test set. The algorithm does not require the use of stress, invasive testing or ionizing radiation, and can be performed at the point of care in resource-constrained environments.

## Materials and methods

### Clinical data

The IDENTIFY trial (NCT03864081) is a prospective, multicenter, non-randomized, selectively blinded, repository study designed to collect and store acquired signals paired with subject meta-data, including outcomes data, from subjects with symptoms of obstructive coronary artery disease. This data was collected for the development, optimization, test and validation of machine-learned algorithms. This repository study has been conducted under study protocol CADF-CIP-001 and is ongoing. Informed consent is required of all subjects participating in this study. The consenting process is compliant with the U.S. Code of Federal Regulations 21 CFR 50, 21 CFR 812, and all other applicable national, local and institutional requirements.

The data used in the development of the CAD algorithm was composed of acquired signals, which were comprised of orthogonal voltage gradient (OVG) and photoplethysmogram (PPG) signals collected simultaneously. These signals were paired with a test result. The signal was collected from subjects with new onset symptoms before undergoing SPECT, coronary CTA, or invasive angiography. Signals were also collected from asymptomatic healthy subjects with no risk factors, which minimizes the likelihood of undetected underlying CAD. Subjects with an unknown or non-determinable CAD status were removed, and signals not passing a signal quality assessment were excluded. All subjects enrolled provided prior consent per the study protocols. Table 1 describes the function of each dataset.

**TABLE 1:** Dataset function.

Dataset	Brief description of dataset	Number of subjects	Function				
			
			Dimensionality reduction	Training	Model confirmation	Internal validation	Blinded validation
CADLAD	Subjects with ICA	653	✓*	✓**			
IDENTIFY Group 4a	Subjects with CCTA, CADRADs 0–3, not referred for further testing	200		✓			
IDENTIFY Group 1	Subjects with ICA, possible pre-existing MI	123			✓		
IDENTIFY Group 3	Subjects with SPECT, negative results	260			✓		
CADHEALTH Group 1	Asymptomatic subjects with no known cardiovascular risk factors, younger	550			✓		
CADHEALTH Group 2	Asymptomatic subjects with no known cardiovascular risk factors, older	108			✓		
IDENTIFY Group 2a	Subjects with ICA	439				✓***	
IDENTIFY Group 4b	Subjects with CCTA, CADRADs 0–3, not referred for further testing	247				✓	
IDENTIFY Group 4V	Subjects with CCTA, CADRADs 0–3, not referred for further testing	182					✓
IDENTIFY Group 2V	Subjects with ICA	783					✓
Total		3,487					

*Only CAD- subjects without any detected lesions nor luminal irregularities (*N* = 207), and CAD + subjects with multi-vessel CAD (*N* = 144), for a total of *N* = 351. **Only CAD + subjects, *N* = 475. ***CAD + subjects used for internal validation testing (*N* = 144).

All data used herein from IDENTIFY Group 2 (ICA) was prior to June 10, 2019 and all from CADFEM Group 4 (CCTA) was prior to December 31 2020. Subjects enrolled in these two groups after these dates were blinded from the algorithm development team and used for validation of the algorithm. These sets are denoted as IDENTIFY Group 2V and IDENTIFY Group 4V in the table.

### Validation population groups

The validation population consists of subjects from IDENTIFY Group 2 (ICA) and IDENTIFY Group 4 (CCTA). Subjects in this set were recruited after the dates presented above.

**FIGURE 1:**
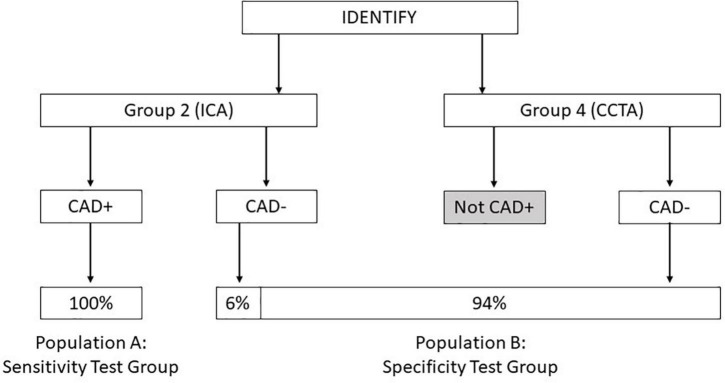
Diagram showing the composition of population A: Sensitivity test group and population B: Specificity test group derived from the IDENTIFY clinical study.

The primary endpoint for validation of the algorithm tested the validation population using two specific test groups, one group for testing sensitivity and one group for testing specificity. The formation of each of these groups is shown in Figure 1.

### Population A: Sensitivity test group

This data set is used to test for device sensitivity in the intended use population.

This data set is comprised of subjects who had presented with no known prior history of CAD and with new onset symptoms suggestive of CAD. These patients were already scheduled for invasive coronary angiography. This data set is built from subjects enrolled into IDENTIFY Group 2 for whom angiography results were available. These subjects are classified as CAD + if any of the following are present:

• A stenosis >50% in the left main coronary artery (LMA).
• A stenosis >70% or fractional flow reserve (FFR) <0.80 or instantaneous wave-free ratio (iFR) <0.89 in the left anterior descending (LAD) artery or any of its distributions (e.g., diagonals, septals).
• A stenosis >70% or FFR <0.80 or iFR <0.89 in the left circumflex (LCX) artery or any of its distributions (e.g., obtuse marginals, acute marginals).
• A stenosis >70% or FFR <0.80 or iFR <0.89 in the Ramus Artery.
• A stenosis >70% or FFR < 0.80 or iFR <0.89 in the right coronary artery (RCA) or any of its distributions (e.g., posterior descending coronary artery (PDA), acute marginals).
• When available, the FFR or iFR assessment result supersedes a percent stenosis result.

### Population B: Specificity test group

This data set is used to test for device specificity in the intended use population.

This data set is comprised of subjects with new onset symptoms suggestive of flow-limiting coronary artery disease at current presentation with no known coronary artery disease. These subjects are determined to be CAD-.

This data set is built from subjects enrolled in the following groups:

∘ IDENTIFY Group 2 subjects who had been referred to a coronary angiography procedure and the results of which were identified as CAD- by assessment of the coronary angiography report.
∘ IDENTIFY Group 4 subjects who had been referred to coronary CTA procedure and were determined by an independent core lab to be negative for significant CAD [Global Institute for Research (GIR), Midlothian, VA, United States]. In order to be classified as negative for significant CAD, these subjects were required to have a CADRADs score of 0–3 and no recommendation for further testing or follow-up.

These groups are weighted in the following proportions for the primary objective analysis:

∘ IDENTIFY Group 2 = 6%, representing the proportion of subjects that would have gone to catheterization (10% of the symptomatic population) and subsequently assessed as CAD- (60% of the catheterization group).
∘ IDENTIFY Group 4 = 94%.

### Machine learning

#### Training

Features are mathematical transforms of the input data that generally result in a number that represents some aspect of the input data. For example, average heart rate over the course of sample collection would be considered one feature. A library of 3298 features has been developed that aims to capture a wide range of aspects of the data that may be useful in identifying cardiac conditions. Some of these features operate on the OVG, some on the PPG, and others on the combination of OVG and PPG signals.

The CAD Model was developed using Elastic Net with cross-validation. Here, the data was divided, while preserving the proportion of diseased subjects to non-diseased subjects, into k subsets, referred to as folds. One fold was kept for testing and the model was trained on remaining data (i.e., k-1 folds), and the AUC evaluated on the testing fold. The process was repeated k times, and on each iteration, a different fold was used for testing. Therefore, each subject was used for testing once, and for training k-1 times. k was configured to be 5. Cross-validation provides a robust estimate of performance through the variation of training and test set membership, which suppresses spurious low or high estimates of test performance (that may be caused by biased training or test sets) and provides an indication of model stability by averaging the test performance across all the folds. To add further rigor by varying how the data is divided into the fivefolds, the process was repeated 100 times.

Elastic Net models were trained using the scikit-learn library in Python 3 on the CAD + subset of CADLAD (*N* = 446) and the CAD- subset of IDENTIFY Group 4a (*N* = 200).

While many machine learning algorithms have been previously used for assessment of cardiac disease, Elastic Net has two advantages that resulted in its selection for this work. First, Elastic Net is particularly effective when the number of features is large compared to the number of training subjects, which is the case both here and in many machine learning applications in the medical domain (14). Increasingly, deep learning methods are being applied, but these require larger volumes of data. Second, the coefficients assigned by Elastic Net to the features allow the feature importance to be easily assessed (15).

To determine the optimal hyperparameters for Elastic Net, a grid search was used with the values provided in Table 2, which resulted in a total of 100 different combinations of hyperparameters. For each set of hyperparameters, stratified fivefold cross validation was performed 100 times. This resulted in a total of 500 individual models for each set of hyperparameters, each trained on 80% of the data (fivefold cross validation) and tested on the 20% remaining data over 500 iterations. The mean AUC of the 500 models on their respective test sets was used for comparing the performance of the various hyperparameters for model selection.

**TABLE 2:** Searched values of Elastic Net hyperparameters.

Hyperparameters	Values
Alpha (**a**)	0, 0.010, 0.100, 0.500, 0.003
l1_ratio (ρ)	0, 0.010, 0.100, 0.500, 3.000
normalize	True or False
fit_intercept	True or False

#### Model selection and confirmation

The set of hyperparameters with the highest mean AUC was selected, and the entire dataset was used to train the model (now referred to as the “CAD Model”). Retraining the model in such a way exposes the model to as much training data as possible, increasing the likelihood that it generalizes to unseen data. The training was fivefold cross validation repeated 300 times. For robustness, the average of the 300 model outputs constitutes the CAD Model output. Since Elastic Net is a linear combination of features, the average model can be thought of as the average of the coefficients and intercept terms from the 300 models. The model with the best mean test AUC of 0.79 uses the hyperparameters of alpha = 0.003, fit_intercept = True, l1_ratio = 0.01, and normalize = True. The hyperparameter values were found to be stable, meaning minimal sensitivity in the model performance given perturbations to the values.

**FIGURE 2:**
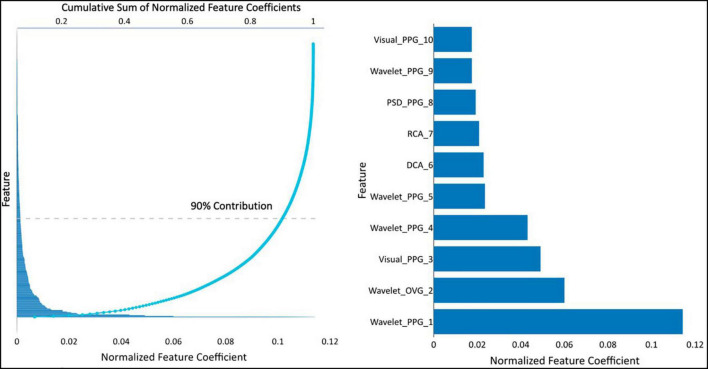
(Left) feature coefficients normalized by feature averages and its cumulative sum, and (right) the normalized feature coefficients for the top 10 features. Visual-PPG — features derived from analyzing the PPG and its first and second derivatives in phase space. Wavelet-PPG — features derived from wavelet analysis of PPG signal. PPG-PSD — features deviates from power spectral density analysis of the PPG signal. RCA, repolarization conduction abnormality; analysis of the ventricular repolarization waveform in band-pass limited frequency ranges. DCA, depolarization conduction abnormality; analysis of the ventricular depolarization waveform in band-pass limited frequency ranges. Wavelet-OVG — features derived from wavelet analysis of OVG signal.

The resultant CAD Model uses 373 of the 3298 available features. The contribution of various features for this model is presented in Figure 2. Of these, 134 of 373 features account for 90% of the prediction.

#### Performance within the internal test set

**FIGURE 3:**
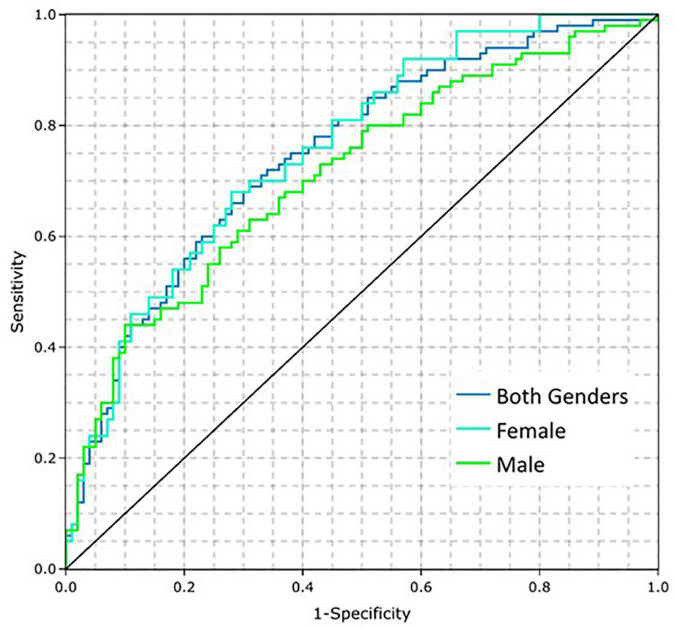
Coronary artery disease (CAD) model performance on the training dataset by gender.

The performance of the CAD Model was tested on 391 subjects from IDENTIFY Group 2 (sensitivity) and IDENTIFY Group 4b (specificity). The CAD Model had an AUC of 0.75 (95% CI: 0.70, 0.80) on both genders. The AUC for males and females were 0.71 (95% CI: 0.64, 0.78) and 0.76 (95% CI: 0.68, 0.84), respectively. Figure 3 shows the receiver operating characteristic (ROC) curves of the model for the entire validation set (both genders, *N* = 391), males (*N* = 193), and females (*N* = 198).

**FIGURE 4:**
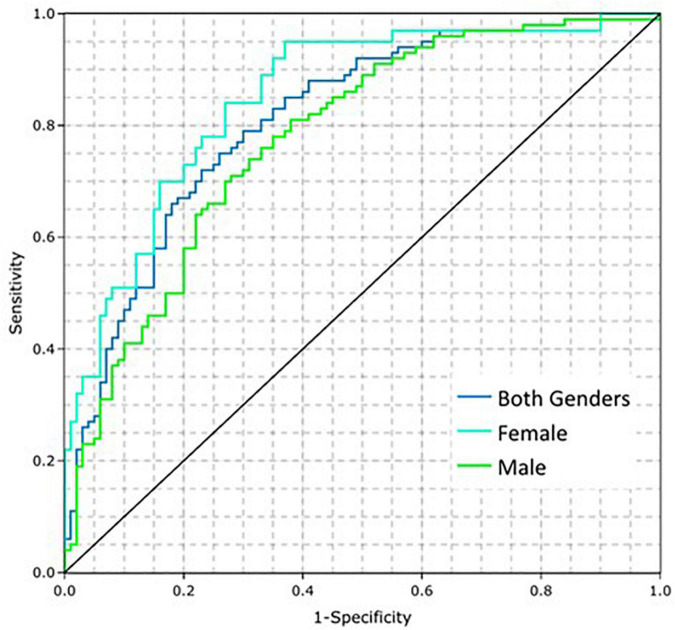
Coronary artery disease (CAD) model performance on the training dataset by gender when adding the influence of age.

The prevalence of CAD is higher in older patients (16), which can be treated as an estimation of pre-test probability that can be integrated into the CAD Model. Such a modification increases bias toward age, but if the intended population is known, then age can be used to modify the predictions. In the present work, a simple approach is used to account for this effect, which is the multiplication of the predictions by age and dividing by 100 (for normalization purposes). Figure 4 shows the ROC curves for the CAD Model including age for both genders, males, and females, which improved the AUCs to 0.81 (95% CI: 0.77, 0.86), 0.78 (95% CI: 0.71, 0.84), and 0.85 (95% CI: 0.78, 0.92), respectively. When modifying the average model using age, setting the sensitivity to 0.78 using a cut point of 0.32171 resulted in a corresponding specificity of 0.69. This threshold was selected prior to running the clinical validation test set. When implemented for validation, this cut point was normalized to zero.

### Null hypotheses for validation

#### Sensitivity

Hypothesis:

H0 = Sensitivity to confirm presence of significant CAD in a symptomatic population (Validation Population A) is less than or equal to 0.66 (sensitivity ≤0.66).

H1 = Sensitivity to confirm presence of significant CAD in a symptomatic population (Validation Population A) is greater than 0.66 (sensitivity >0.66).

Validation Population A consists entirely of those subjects who were determined CAD positive by coronary angiogram, following an initial symptomatic presentation. Population A therefore directly aligns with the population required to calculate sensitivity in this context and hence is the appropriate population on which to test the CAD algorithm sensitivity performance.

Furthermore, the entire spectrum of disease is present in Validation Population A, from subjects who went to angiography but had zero lesions, through those with one or more lesions that did not meet the ACC definition for significant CAD, to those with one or more lesions that do meet the ACC definition for significant CAD. Table 3 shows the set of subjects used for development of the algorithm, enrolled under the same protocol as the validation population. It can be seen that approximately one third (32%) of subjects who go to coronary angiography have zero lesions, one third (33%) have one or more lesions, none of which meet the ACC definition of significant CAD, and one third (35%) of subjects have one or more lesion that does meet the ACC definition of significant CAD. Of those subjects with at least one lesion, 90% have a lesion in the LAD, 59% have a lesion in the LCX and 67% have a lesion in the RCA. Given that the same protocol was used to recruit the Validation Population, it is expected that the Validation Population will similarly represent the entire spectrum of disease.

**TABLE 3:** Spectrum of disease observed in the population used for algorithm development.

	Number of subjects	Percentage
**Breakdown by lesion severity (All Locations)**		
Total Size of IDENTIFY Group 2 used in development	487	100%
Subjects with no lesions	156	32%
Subjects with at least one lesion	331	68%
Subjects with most severe lesion not meeting ACC definition	159	33%
Subjects with at least one lesion meeting ACC definition	172	35%
**Breakdown by lesion location (Any Severity)**		
IDENTIFY Group 2 subjects with at least one lesion	331	100%
LAD	298	90%
LCX	195	59%
RCA	222	67%

A normal approximation test (using the estimate of sensitivity and the estimate of the variance of the estimate of sensitivity) is used to test the null hypothesis that the true sensitivity is less than or equal to the performance goal vs. the alternative hypothesis that it is greater than the performance goal using a one-sided α = 0.05. This is the statistical equivalent to showing that the one-sided 95% lower confidence bound is greater than the performance goal. Let n denote the sample size for CAD positive subjects and S denote the estimates of sensitivity. Then, the estimate of the variance of sensitivity is calculated using the following formula:

Estimate of variance of estimate of sensitivity = (S) (1-S)/n.

#### Specificity

Hypothesis:

H0 = Specificity to confirm absence of significant CAD in a symptomatic population (Validation Population B) is less than or equal to 0.60 (Specificity ≤0.60).

H1 = Specificity to confirm absence of significant CAD in a symptomatic population (Validation Population B) is greater than 0.60 (Specificity >0.60).

Specificity solely requires subjects who are deemed to be truly negative for CAD to be calculated. In current clinical practice, a large proportion, approximately 90%, of subjects presenting with new onset symptoms associated with CAD are determined to not have CAD without undergoing coronary angiography (4). Consequently, in order to mirror the use of these testing modalities in clinical practice, 94% of Validation Population B is comprised of subjects assessed to be CAD negative using coronary CTA.

The remaining 6% of subjects in Validation Population B represents the set of subjects who were referred to coronary angiogram and assessed in that procedure to be CAD-. Although these subjects are collected through a different arm of the IDENTIFY study (Group 2) they are representative of the subset of patients referred to coronary angiography through the application of standard of care. Consequently, it is appropriate to use this subset of subjects as representative of those subjects that would have gone on to coronary angiogram in Group 4 and subsequently be determined to be CAD-. Mixing in this way results in a group that is representative of the symptomatic population determined to be negative per the current standard of care. Validation Population B is therefore suitable for assessment of specificity in this context.

A normal approximation test (using the estimate of specificity and the estimate of the variance of the estimate of specificity given below) will be used to test the null hypothesis that the true specificity is less than or equal to the performance goal vs. the alternative hypothesis that it is greater than the performance goal using a one-sided α = 0.05. This is the statistical equivalent to showing that the one-sided 95% lower confidence bound is greater than the performance goal.

Let v_2_ and v_4_ represent the observed relative frequencies for Groups 2 and 4, respectively, of CAD negative subjects. Let n_2_ and n_4_ denote the sample sizes for Groups 2 and 4, respectively. Let S_2_ and S_4_ denote the estimates of specificity for Groups 2 and 4, respectively. Then, the estimate of specificity and the estimate of the variance of specificity are calculated using the following formulae:


Estimateofspecificity=v(S)22+v(S)44

Estimateofvarianceofestimateofspecificity=()2(S)22(1-S)2/n+2(v)4(S)42(1-S)4/n4

The formula for the estimate of the variance of the estimate of specificity was derived using the formula for the estimated variance of the sum of independent (weighted) proportions. Per Figure 1, v_2_ = 0.06 and v_4_ = 0.94.

## Results

**FIGURE 5:**
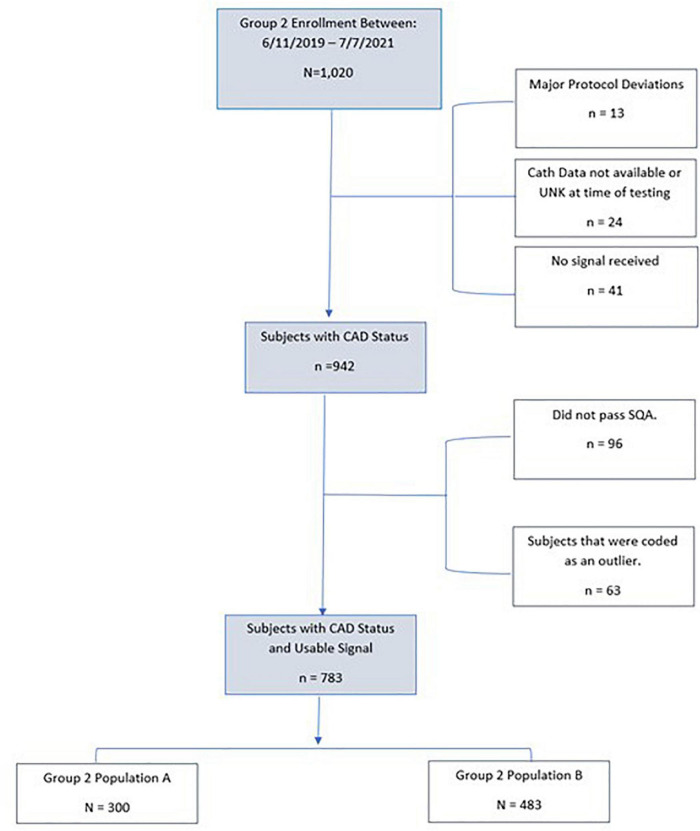
Consort diagram of Group 2 (Population A and Population B) validation subjects.

As tabulated in Figure 5, 1,020 subjects were enrolled into the validation set with a referral for invasive coronary angiography. Of these, 41 subjects (4%) did not have a received signal. Additionally, there were 24 subjects (2.3%) that either did not have invasive coronary angiography or results were not available at the time of testing. Thirteen subjects had a major protocol deviation. Major deviations were subjects with catheterization greater than 7 days from signal collection or were enrolled and not found to meet study entrance criteria.

The remaining subjects (*n* = 942) enrolled in the Group 2 Population A and Population B met all inclusion and no exclusion criteria, and had no major protocol violations. Of this subset, 10% (*n* = 96) had signals received that did not have acceptable signal quality. An additional 6.7% (*n* = 63) had a passing signal quality but were deemed an outlier by the outlier detection method. The remaining 783 subjects were used in this validation set. Group 2, population A, consisted of 300 (78%) subjects that were positive for significant CAD and used for sensitivity testing. Group 2, population B consisted of 483 (61.7%) subjects that were used for specificity testing.

**FIGURE 6:**
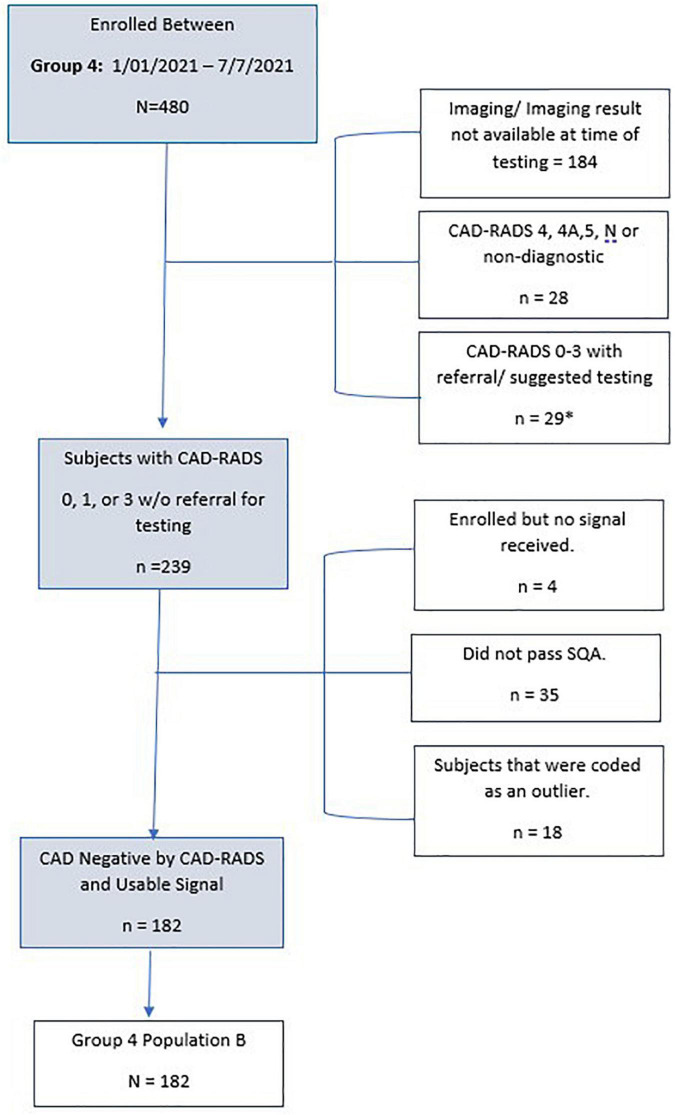
Consort Diagram of Group 4 validation subjects for specificity. *n = 3 with unknown treatment referral.

As tabulated in Figure 6, 480 subjects were enrolled in IDENTIFY Group 4. Of these, 184 subjects did not have the imaging result completed at the time of the analysis. Additionally, there were 28 subjects (6%) where they were either CAD-RADS 4, 4A, 5 (likely positive or high risk for coronary disease) or had images that were deemed non-diagnostic. Of the remaining 276 that were CAD-RADS 0–3 (no significant coronary artery disease), 29 were referred for additional testing, additional testing was suggested or the referral status was unknown (*n* = 3).

The remaining subjects (*n* = 239) enrolled in Group 4 Population B met all inclusion and no exclusion criteria, had no major protocol violations and only 4 did not have a signal received. This is typically due to connectivity issues or improper use of the device i.e., not keeping the device charged. Of the remaining subset, 15% (*n* = 35) had signals received that did not have acceptable signal quality. There were an additional 7.5% (*n* = 18) that had a passing signal quality but were deemed an outlier by the outlier detection method. The remaining 182 subjects were used for CAD validation for specificity. Table 4 details the validation population by Group and population.

**TABLE 4:** Demographic breakdown of the validation populations A and B.

Variable	Statistics	Population A (*N* = 300)	Population B (*N* = 665)	Total (*N* = 965)
Age at time of consent (years)	*n*	300	664*	964*
	Mean	65.5	59.5	61.4
	Median	66.0	60.0	63.0
	SD	8.62	10.91	10.61
	Minimum–Maximum	40–90	30–86	30–90
**Age (years)**				
<65	*n* (%)	125 (41.7)	418 (63.0)	543 (56.3)
> = 65	*n* (%)	175 (58.3)	246 (37.0)	421 (43.7)
Gender				
Male	*n* (%)	202 (67.3)	307 (46.2)	509 (52.7)
Female	*n* (%)	98 (32.7)	358 (53.8)	456 (47.3)
**Ethnicity**				
Hispanic or Latino	*n* (%)	5 (1.7)	6 (0.9)	11 (1.1)
Not hispanic or latino	*n* (%)	293 (98.3)	659 (99.1)	952 (98.9)
**Race**				
American Indian or Alaska Native	*n* (%)	3 (1.0)	1 (0.2)	4 (0.4)
Asian	*n* (%)	1 (0.3)	2 (0.3)	3 (0.3)
Black or African American	*n* (%)	28 (9.3)	131 (19.7)	159 (16.5)
Native Hawaiian or Other Pacific Islander	*n* (%)	0 (0.0)	0 (0.0)	0 (0.0)
White/Caucasian	*n* (%)	266 (88.7)	527 (79.2)	793 (82.2)
Other	*n* (%)	0 (0.0)	4 (0.6)	4 (0.4)
Prefer not to answer	*n* (%)	2 (0.7)	0 (0.0)	2 (0.2)
Weight (kg)	*N*	300	665	965
	Mean	91.8	96.0	94.7
	Median	91.5	94.3	92.5
	SD	18.30	22.89	21.65
	Minimum–Maximum	50–174	44–174	44–174
Height (cm)	*N*	299	665	964
	Mean	171.9	170.2	170.8
	Median	172.7	170.2	170.2
	SD	9.67	10.48	10.26
	Minimum–Maximum	137–198	137–198	137–198
BMI (kg/m^2^)	*N*	299	665	964
	Mean	31.0	33.1	32.5
	Median	30.6	32.1	31.6
	SD	5.87	7.45	7.06
	Minimum–Maximum	19–64	14–64	14–64
**BMI Categories (kg/m^2^)**				
<30	*n* (%)	131 (43.8)	250 (37.6)	381 (39.5)
≥ 30	*n* (%)	168 (56.2)	415 (62.4)	583 (60.5)

*Age was not reported for one individual in the EDC, but the age was present in the signal data file.

Following removal of subjects for major protocol deviations, failing the signal quality score, and following the outlier detection module, Population A (sensitivity) consisted of 300 subjects, and Population B (specificity) consisted of 665 subjects, of whom 483 were assessed to be CAD negative using ICA, and 182 using coronary CTA.

Table 5 shows that the null hypothesis is rejected for both sensitivity and specificity tests. The algorithm therefore passes the pre-defined endpoints at the 95% confidence level. The ROC-AUC for the algorithm across the validation set is 0.75 (0.73, 0.78).

**TABLE 5:** Results of the machine learned algorithm when applied to the validation population.

	Predicted CAD status (CAD Add-On)					
	
	Population A			Population B		
		
Confirmed CAD status	PredCADPos	PredCADNeg	Total	PredCADPos	PredCADNeg	Total
CAD positive	218	82	300	0	0	0
CAD negative	0	0	0	334	331	665
Total	218	82	300	334	331	665
Co-primary endpoint: Sensitivity (Population A)				Estimate	0.73	
				95% LCB	0.68	
				*p*-value	0.048	
Co-primary endpoint: Specificity (Population B)				Estimate	0.68	
				95% LCB	0.62	
				*p*-value	0.0056	

Subgroup analyses of sensitivity and specificity performances were performed for major subgroups, with the results shown in Table 6. Table 6 shows that there are significant differences (*p* < 0.01) in sensitivity between females and males, sensitivity and specificity between older (age ≥ 65 years) and younger (age <65 years) subjects, specificity between hypertensive and non-hypertensive subjects, and in specificity between hyperlipidemic and non-hyperlipidemic subjects.

**TABLE 6:** Subgroup analyses of sensitivity and specificity performances in the validation populations.

	Predicted CAD status (CAD Add-On)			
	
	Population A		Population B	
		
Subgroups	(*N* = 300)	*P*-value (1)	(*N* = 665)	*P*-value (2)
Female	0.62	0.0048	0.67	0.2737
Male	0.78		0.71	
BMI ≥ 30 kg/m^^2^	0.74	0.6918	0.72	0.0374
BMI < 30 kg/m^^2^	0.72		0.63	
Age ≥ 65 years	0.87	<0.0001	0.22	<0.0001
Age < 65 years	0.52		0.85	
Diabetic	0.73	0.9191	0.62	0.1050
Non-diabetic	0.73		0.69	
Hypertensive	0.75	0.0261	0.62	0.0004
Non-hypertensive	0.61		0.77	
Hyperlipidemic	0.72	0.5035	0.61	<0.0001
Non-hyperlipidemic	0.76		0.79	
Smoker (Past or Present)	0.71	0.3556	0.68	0.9009
Non-smoker	0.75		0.68	

(1) *p*-value from two-sided normal approximation test, testing the null hypothesis that the true sensitivities are equal for the two subgroups vs. the alternative hypothesis that they are not equal. (2) *p*-value from two-sided normal approximation test, testing the null hypothesis that the true specificities are equal for the two subgroups vs. the alternative hypothesis that they are not equal.

The above results demonstrate that the CAD algorithm performed as expected in the validation set. The sensitivity and specificity at the pre-specified cut point was within the 95% confidence bounds of the ROC curves created when assessing the algorithm during the training phase. Furthermore, the algorithm met the pre-specified end points for demonstrating clinically acceptable performance in the validation set.

When assessing the performance of machine-learned algorithms, it is crucial to consider the intrinsic and extrinsic biases that may affect the source data, and potentially become embedded in the algorithm (17). Common issues that may affect such algorithms include the heterogeneity between methodologies for performing the reference standard tests between institutions, and also by potential differences in disease prevalence in different geographies. The validation population presented here is comprised of subjects that reflect trends seen in the United States population with respect to prevalence of cardiovascular disease by state and includes those with higher prevalence (New York, Oklahoma, Texas, Mississippi, Louisiana), moderate (South Carolina, Florida) and those with lower prevalence comparatively (North Carolina, Kansas, Georgia, Nebraska) (18). Thus, the validation population tested is expected to be representative of the intended use population. Furthermore, the validation set was sourced from 16 distinct sites, including both hospital and office settings, to further mitigate the potential for any site-based bias to confound the algorithm performance.

A second potential pitfall in generating machine-learned algorithms is that of overfitting of the data used during training (17). The results presented here are from a blinded validation set and not training data and hence have been shown to generalize beyond the training set.

Thirdly, it is important to consider whether there are any demographic differences in performance. In particular, historically, women and minority groups are under-represented in cardiology (17). The results presented here do show a lower performance of the algorithm in females. This is commensurate with that observed in myocardial perfusion imaging and may also be related to the smaller size of the left ventricle. Further work in this area to improve this performance will require more females to be brought into the learning set, which is difficult given the relatively low numbers of females presenting with significant CAD. Also, there may be specific features that perform better, or indeed worse, on female subjects that if identified may help to improve performance in this group. In the validation set there were over 15% Black or African American subjects, with no difference observed in algorithm performance between this sub-group and the overall performance.

## Discussion

**FIGURE 7:**
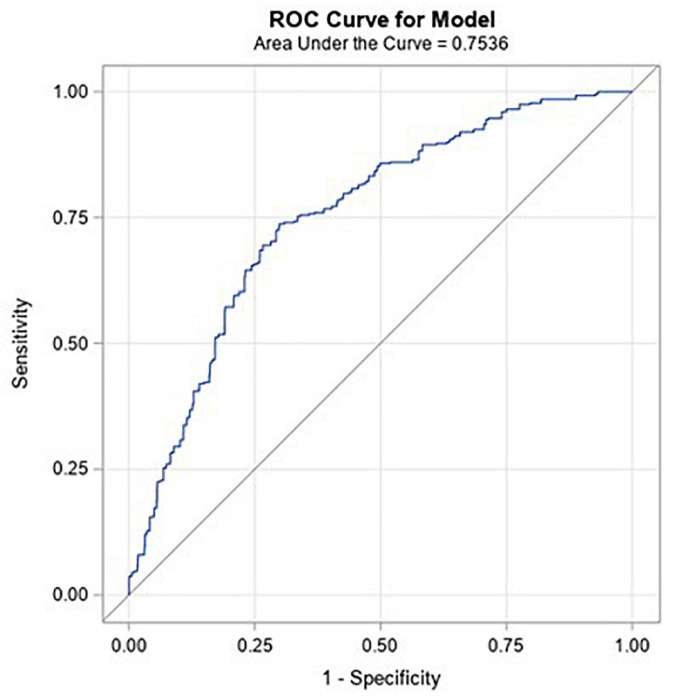
ROC curve for the model against the validation population.

It is difficult to directly compare performance of tests, such as coronary CTA and SPECT, as they operate with very different skews toward sensitivity and specificity. Similarly, it is difficult to directly compare the results of the presented algorithm to these tests. However, an advantage of a machine learned algorithm such as this is that the complete ROC curve is available, shown in Figure 7. Given the availability of the ROC curve, it is possible to identify points on the curve which may provide similar skew toward sensitivity or specificity as other diagnostic tests. For example, coronary CTA may be used to identify individuals for whom significant CAD is unlikely, i.e., a rule-out test. Such a test would have a very high negative predictive value: Assuming a pre-test disease prevalence of 0.04, and the sensitivity of 0.93 (0.89–0.96) and specificity of 0.53 (0.370.68) for functionally significant CAD presented by Knuuti, et al. (5), the NPV for coronary CTA would be 0.99. There is a point on the ROC curve (cut point of −0.07) for the machine learned algorithm presented here that has the same NPV. At this point, the sensitivity is 0.89 and the specificity is 0.42. These are within the 95% confidence bounds for the performance of coronary CTA reported in the Knuuti analysis.

SPECT is a functional test with a sensitivity of 0.73 (0.62–0.82), specificity of 0.83 (0.71–0.90) (5), and hence a higher PPV (0.15, assuming the same pre-test prevalence of 0.04), that may be used to identify individuals to progress to more invasive tests, such as ICA. There is a point on the ROC curve for the CAD algorithm (cut point of 0.1) presented here that maximizes PPV at 0.12. At this point, the sensitivity is 0.39 and specificity is 0.88. These values are below the 95% lower confidence bound of SPECT for sensitivity and within the 95% confidence bounds for specificity (5). However, the algorithm could still be used in a similar manner, to rule in individuals for further testing.

**FIGURE 8:**
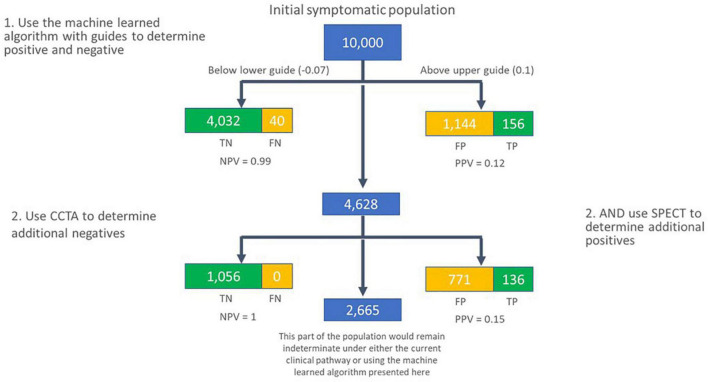
Flow of subjects in a hypothetical population of 10,000 individuals with new onset symptoms of CAD, assuming a pre-test prevalence of 0.04. In the first pass, the machine learned algorithm presented here is used to call individuals as negative for significant CAD if their score is lower than –0.07, and likely positive for functionally significant CAD if their score is greater than 0.1. The group in the middle are secondarily assessed using coronary CTA and SPECT to determine additional subjects that are unlikely to have significant CAD (coronary CTA), or likely to have significant CAD (SPECT). TN, true negative; FN, false negative; TP, true positive; FP, false positive; NPV, negative predictive value; PPV, positive predictive value.

If a physician group were to interpret the algorithm scores in this way, with a lower guide of −0.07, and an upper guide of 0.1, across a large, hypothetical population of 10,000 individuals, with an assumed pre-test prevalence of 0.04, the true and false calls would be as shown in Figure 8. A limitation of this work is that the multiple cut-points used in this analysis were not pre-specified. In future work, pre-specification of these cut-points, and associated hypotheses would allow for the approach to be validated against a different test set.

The performance of the algorithm in correctly identifying significant CAD in females (sensitivity) is significantly lower than that for males (*p* = 0.0048). A similar issue exists with current tests too. In one study, SPECT performance decreased significantly as the volume of the left ventricle decreased (19). This decrease in left ventricular volume was driven by gender, with the smaller ventricles observed in females (*p* < 0.0001) (19). The performance of SPECT reported in this group was a sensitivity of 0.43 and a specificity of 0.90 (19). Furthermore, in a study of anatomically defined significant CAD (lesion ≥ 50%) vs. functionally defined significant CAD (FFR <0.8), females were identified as having an increased likelihood of an anatomically derived significant CAD not matching the functional definition (20). This could be due to physiological differences in how lesions impact arterial blood flow in female subjects, such as differences in the elasticity of the arterial walls. A limitation of the current work is that there are fewer female CAD positive subjects available than male CAD positive subjects, at a ratio of approximately 1:2 (see Table 4). The yield of CAD positive subjects from those undergoing ICA is also low, at around 35% (see Table 3). Consequently, for every subject enrolled in IDENTIFY Group 2, only 1 in 10 will be female CAD positive. This impacts training and testing of the algorithm: The algorithm would likely be improved by exposure to larger numbers of female CAD positive subjects. It would also be desirable to power the clinical performance test to be gender specific. This would require recruitment of a large number of subjects; for example, around 175 subjects might be required to power the end points presented here, requiring 1,750 subjects to be recruited to the validation test population, or more than twice the number available for the currently presented work. Also, this would not include the transfer of larger numbers of CAD positive females to the training set. As the IDENTIFY study is ongoing, such numbers may eventually be available for future work in this area.

When considering the change in probability of disease when applying this CAD algorithm, we can see that the likelihood ratios at the validation performance are LR + = 2.28 and LR− = 0.41. The likelihood ratios for coronary CTA are LR + = 1.97 and LR− = 0.13; and for SPECT are LR + = 4.21 and LR− = 0.33 (5). As expected from the assessment of sensitivity and specificity, the likelihood ratios between these three tests are also comparable, with the CAD algorithm test performance at the pre-specified cut-point lying in between those of coronary CTA and SPECT.

Major meta-analyses cited in the ACC guidelines expose the difficulty in establishing the precise accuracy of different diagnostic tests (1). For example, in a repeatedly referenced exercise ECG meta-analysis involving 24,074 patients from 147 studies of symptomatic patients who underwent exercise ECG and invasive coronary angiography, there was remarkable variation in the exercise ECG sensitivity and specificity; the mean sensitivity was 68% but ranged from 23 to 100%, and the mean specificity was 77% but ranged from 17 to 100% (1, 21). Similarly, in a review of 13 studies utilizing SPECT in 1,323 patients, the sensitivity was 85% but ranged from 76 to 91%, and the mean specificity was 66% with a range of 54–77% (22). Additionally, the reference standard for these was anatomically, not functionally, significant CAD.

The definition of “significant CAD” may be different in different studies. For example, a common measure of significant anatomical CAD would be an occlusion of greater than 50% in a major artery. However, ACC guidelines classify a significant lesion, for the purpose of intervention using a stent, as any lesion greater than 70%. Increasingly fractional flow reserve (FFR) is used as a functional measure of the impact of CAD, whereby an FFR ≤0.8 is classed as significant (5). In the review referred to above, (22) the sensitivity and specificity may have been reported for an anatomic occlusion of greater than 50%, or greater than 70%. If multiple definitions or multiple results from different observers were reported, then the review reported the performance associated with the greatest sensitivity (22). Indeed, given that the specific morphology of a lesion may have a significant impact on blood flow beyond that of solely the percentage of the artery occluded, it is perhaps not surprising that an anatomically significant occlusion of greater than 50% occlusion may only be predictive of a functionally significant occlusion (FFR ≤0.8) 68% of the time (mean sensitivity of 68%, ranging from 60 to 75%, and mean specificity of 73%, ranging from 55 to 86%) (5). In that same analysis, the performance of SPECT against a functional definition of significant CAD (FFR ≤0.8) was reported as a sensitivity of 73% (62–82), and a specificity of 83% (71–90) (5).

Park et al. (20) analyzed a set of 1,066 lesions in major arteries (excluding the LMCA) for which both anatomic and FFR assessments were performed during ICA. The concordance between the 50% anatomic threshold and the 0.8 FFR threshold was low, with a 57% mismatch wherein the anatomic definition classed the lesion as significant, but the FFR was not significant. Additionally, there was a 16% reverse mismatch wherein the FFR measurement was significant, but the blockage was less than 50%. Park et al. did not perform an assessment at the 70% anatomic threshold, but an analysis of their data, presented in Figure 1A of their paper shows that the concordance is higher. When applying an anatomic threshold of 70% to the data presented, there are 21 lesions that have an FFR >0.8, and 57 lesions that have an FFR <0.8. In the terms introduced in the paper, there is a 27% mismatch, with a 28% reverse mismatch. In terms of sensitivity and specificity, this would imply that in this cohort, an anatomic threshold of 70% is 73% sensitive and 68% specific in terms of identifying CAD that is functionally significant when determined by FFR. This happens to be the same numeric performance as the CAD algorithm presented here in detecting significant CAD, when an anatomic threshold of 70% or FFR of less 0.8 is used as the reference standard. One limitation of this current work is that CAD positive subjects are defined either by FFR, iFR or, if neither is available, the anatomic definition. The inherent uncertainty in the label that this introduces may be hampering training and ultimately impacting performance. Collecting large numbers of subjects with FFR is difficult as many centers do not perform this as standard on all lesions. Further research on this topic would, however, be beneficial.

Given that multiple trials have demonstrated broad ranges for sensitivity and specificity when attempting to detect CAD and that this depends on the test modality used, (1, 21, 22) it becomes relevant to assess the safety of applying any of these tests. It is well established that when evaluating patients presenting with new onset chest pain or other symptoms of obstructive CAD, regardless of whether functional testing (exercise ECG, stress echocardiography, myocardial perfusion imaging) or coronary CTA is the first evaluation, 90% will have negative findings (4). In this scenario, a negative test portends an excellent long-term outcome (NPV >96%) (4). In addition, patients with an initial positive test almost always have further evaluation, typically with angiography. In those individuals who ultimately have angiography, only 32–42% have significant CAD (≥70% obstruction) (4, 23). Only 3–4% of the patients initially presenting with symptoms of obstructive CAD are ultimately identified as having significant CAD (4).

To the authors’ knowledge, there are no systems available at the point of care using synchronous OVG and PPG signals as the basis for a machine learned assessment of the likelihood of CAD. The following two related assessments have been identified: 1) The CAD score V3 algorithm is based on a combination of acoustic signals and clinical risk factors (24). This algorithm had a validation test performance of predicting the likelihood of significant CAD (defined by FFR or anatomic lesion ≥90%) of sensitivity = 79% (69–87) and specificity = 52% (49–56), with an AUC of 0.7 (0.64–0.76) (24). The algorithm included gender, age and hypertension (defined as systolic blood pressure ≥140 mm Hg or receiving antihypertension medicine) as clinical risk factors. Without these, the AUC of the acoustic component alone was reported as 0.63 (0.59–0.68), with the output of the acoustic component being significantly higher for men than for women (24) and thus more likely to correctly identify CAD positive men than women. 2) A blood-based gene expression algorithm for the assessment of anatomically significant CAD (lesions ≥50%) has been presented with an AUC of 0.7 (0.68–0.72) (25). When the cut-point was set to classify 20% of the validation set as positive, the reported sensitivity was 85% with a specificity of 43%. Performance of the algorithm was similar in both men and women, with AUCs of 0.66 and 0.65, respectively (25). The AUC performance of the algorithm presented here, 0.75 (0.73, 0.78), compares favorably with both of these systems.

Accurate identification of patients with coronary artery disease is critical in clinical medicine. From a clinical standpoint, although the risk of significant CAD is low, it is important to detect the group that is at higher risk. The clinical utilization of available testing modalities is based on the facts that the NPV of these tests is high and individuals with a positive finding will undergo additional testing. With this approach, risk to the patient is best mitigated. When presented with the data on which to make such determinations, such as the ROC curve for a diagnostic test, or similar plots, physicians can use that information to make informed decisions with a single test that would be difficult with either coronary CTA or SPECT alone. For example, the algorithm presented here could be used as a front-line diagnostic test to rule out individuals unlikely to have significant CAD when their score is at or below −0.07, or to rule in individuals for further testing, when their score is at or above 0.1. Individuals in the intermediate range may also be considered for further testing, perhaps using coronary CTA or SPECT. The use of a front-line test in this manner could potentially avoid significant downstream testing, thereby avoiding exposure to ionizing radiation and the significant cost, time and inconvenience incurred in standard of care assessment.

In conclusion, the performance of the machine learned algorithm presented here is comparable to current commonly used tertiary center testing for CAD. The use of multiple cut points on the receiver operating characteristic curve allows for a test which has the negative predictive value of coronary CTA and a positive predictive value approaching that of myocardial perfusion imaging. As the system may be used in a standard office setting, it may address the need for a non-invasive, no radiation, no stress, front line test, and hence offer significant advantages to the patient, their physician, and healthcare system.

## Data availability statement

The datasets presented in this article are not readily available because the dataset is proprietary to CorVista Health. Requests to access the datasets should be directed to IS, ishadforth@corvista.com.

## Ethics statement

The algorithm presented here is an investigational device. Limited by United States law to investigational use. The studies involving human participants were reviewed and approved by This IDENTIFY protocol and related documents were approved by local or regional Institutional Review Boards (IRBs) as per local policies before the study is initiated at the site. A copy of the study-specific IRB approval letter and the IRB-approved Informed Consent Form was sent to the Sponsor prior to initiating the study activities. Each Investigator was responsible for keeping the IRB advised of the progress of the study as deemed appropriate and in alignment with IRB guidelines and requirements (at minimum, once a year during the course of the study). The Investigator kept the IRB and Sponsor informed of any adverse events. Ethical Conduct of the Study this exempt IDE study was conducted according to the principles of Good Clinical Practice ICH E6 and relevant requirements of 21 CFR 812 and 21 CFR Part 56 to ensure valid scientific evidence is obtained per the recommendation in FDA Guidance for Industry and FDA Staff titled “*in Vitro* Diagnostic (IVD) Device Studies -Frequently Asked Questions (issued June 25, 2010).” Institutional Review Board (IRB) approval with assessment supporting the determination the study is non-significant risk (NSR) and that suitability of the device(s) for human use have been demonstrated under the auspices of established R&D test plans, product labeling, informed consent, study conduct (monitoring, records and reports for the Sponsor and Investigator); and prohibitions. The patients/participants provided their written informed consent to participate in this study.

## Author contributions

HG, IS, WS, and MGR: concept and design. IS, MR, TB, FF, AK, EL, HG, and WS: drafting of the manuscript. HG, EL, TB, AK, and FF: statistical analysis. TB, FF, AK, IS, WS, and MGR: literature search. WS: obtained funding. HG, WS, and TB: administrative, technical, or material support. HG and WS: supervision. MGR: final approval of the version to be published. All authors acquisition, analysis, or interpretation of data, and critical revision of the manuscript for important intellectual content.
